# The MOVE-trial: Monocryl® vs. Vicryl Rapide™ for skin repair in mediolateral episiotomies: a randomized controlled trial

**DOI:** 10.1186/s12884-017-1545-8

**Published:** 2017-10-16

**Authors:** Roeland Odijk, Bernadette Hennipman, Melek Rousian, Khadija Madani, Marja Dijksterhuis, Jan Willem de Leeuw, Arjan van Hof

**Affiliations:** 1 Department of Obstetrics and Gynecology, Ikazia Hospital, Rotterdam, The Netherlands; 2 Department of Obstetrics and Gynecology, Amphia Hospital, Langendijk 75, 4819 EV Breda, The Netherlands; 3 GGZ Central, Centre for mental healthcare, Almere, The Netherlands; 4 Department of Obstetrics and Gynecology, Erasmus MC, University Medical Centre, Rotterdam, The Netherlands; 5 Department of Obstetrics and Gynecology, Groene-Hart Hospital, Gouda, The Netherlands; 6 Department of Obstetrics and Gynecology, van Weel-Bethesda Hospital, Dirksland, The Netherlands

**Keywords:** Episiotomy; randomized controlled trial; polyglactin 910, Poliglecaprone 25, Pain, Wound dehiscence

## Abstract

**Background:**

Previous studies have shown that complaints after episiotomy repair depend on the method and material used for repair. The objective of our study was to determine which of two frequently used suture materials, Monocryl® (poliglecaprone 25) and Vicryl Rapide™ (polyglactin 910), is superior for intracutaneous closure of the skin in mediolateral episiotomies.

**Methods:**

In a randomized controlled trial performed in a teaching hospital in the Netherlands between 2010 and 2013 250 primiparous women with uncomplicated mediolateral episiotomies were randomly allocated to intracutaneous skin closure with either Monocryl® or Vicryl Rapide™. All other layers were sutured with Vicryl 2-0 and Vicryl 0 in both groups. Pain scores and complications were documented using questionnaires during the first three months post partum. The primary outcome was pain 10 days after delivery in sitting position established by Visual Analogous Scale (VAS). Secondary outcomes were pain scores at different time points and reported complications such as infections, dehiscence and dyspareunia one day, 10 days, six weeks and three months after delivery.

**Results:**

Of 250 allocated women 54% returned questionnaires. No statistical difference was found between both groups for the primary outcome (VAS 2,8 (95% CI 2,18-3,44) vs. VAS 2,5 (95% CI 2,00-2,98), *p* = 0,43). With regard to secondary outcomes only self-reported dehiscence was significantly different, favouring Monocryl® (10% vs. 25%, *p* = 0.016).

**Conclusions:**

Use of Monocryl® 3-0 and Vicryl Rapide™ 3-0 for intracutaneous closure of the skin after mediolateral episiotomy leads to equal pain scores ten days after delivery and therefore both materials may be considered for this use. Monocryl® 3-0 might be favourable over Vicryl Rapide™ 3-0 due to less self-reported dehiscence after intracutaneous closure of the skin in mediolateral episiotomies.

**Trial registration:**

The trial was retrospectively registered under trial nr. ISRCTN29869308 on 20-04-2016.

**Electronic supplementary material:**

The online version of this article (10.1186/s12884-017-1545-8) contains supplementary material, which is available to authorized users.

## Background

Sixty to 90 % of women suffer perineal trauma during vaginal delivery with sixty to 70 % of these injuries requiring immediate repair [1–3]. Episiotomies are the most frequently performed operative procedure during delivery. In the Netherlands in 47% of primiparous and 15% of multiparous women an episiotomy is performed during delivery [4, 5]. Ninety-two percent of women delivered with an episiotomy will have complaints of perineal pain in the postpartum period and approximately 20% of women still have complaints after three months [6] and 10% even up to 18 months [7].

After episiotomy repair, complaints like pain and dyspareunia may interfere with daily lives of women. The technique and material used in the repair may influence the intensity and duration of these complaints [8–11]. Previous trials have shown that interrupted suturing of the skin causes more pain when compared to continuous intracutaneous suturing [12]. Similarly, it was shown that synthetic materials cause a reduction in complaints when compared to catgut [13, 14]. To date, only two trials have compared a resorbable monofilament and a resorbable multifilament synthetic material for the repair of episiotomies [15, 16].

To date, few RCTs comparing Monocryl® and Vicryl Rapide™ have been performed and no trials have looked specifically at use of both of these materials for skin closure in mediolateral episiotomies [17]. Both materials are frequently used for this purpose in the Netherlands, but differ substantially in their properties [18, 19].

It has been shown that Monocryl® causes minimal tissue reaction due to the fact that as a monofilament material it has a small surface. It is completely resorbed in approximately 120 days. After 14 days it still holds 25% of its tensile strength [20].

Vicryl Rapide™ is a multifilament material. Due to its braided structure, its surface is larger and it might have niches in which bacteria cannot easily be reached by cells of the immune system [21]. Vicryl Rapide™ is completely resorbed in 42 days and has no tensile strength after 14 days.

The objective of our randomized controlled trial was to investigate whether Monocryl® or Vicryl Rapide™ is superior with regard to pain after repair, wound infection and dehiscence after the suturing of the skin after a vaginal delivery with a mediolateral episiotomy in primiparous women.

## Methods

Our trial was performed between 2010 and 2013 at the maternity ward of a single general hospital in Rotterdam, the Netherlands. On average this centre provides care for almost 2000 medium and high-risk deliveries per year. The population attending this hospital is known to have a wide socioeconomical and ethnical range.

Only primiparous women were included in the trial to exclude effects of previous birth trauma and minimise variability. Nulliparous women were informed about the study between the 34th and 36th week of pregnancy. If during delivery a mediolateral episiotomy was performed women were asked to participate in the study.

Women who met the inclusion criteria were randomly allocated to suturing of the skin with Monocryl® or Vicryl Rapide™ after informed consent was obtained. Random assignment took place using opaque envelopes present on the delivery ward. The suture material was included in the opaque envelope containing the questionnaires. The trial was single blinded.

Inclusion criteria were defined as primiparous women with an uncomplicated episiotomy after a vaginal delivery. Both women with a spontaneous and an operative delivery were included. Uncomplicated episiotomy was defined as a mediolateral episiotomy with no additional labial, contralateral vaginal wall or perineal ruptures.

Exclusion criteria were defined as women under 18 years of age or unable to understand the written information about the study. Women with a coagulopathy or an impaired immune system were excluded. Postpartum hemorrhage, prohibiting sufficient time for inclusion, was another exclusion criterium.

Postpartum suturing was performed in the delivery room by the attending midwife or obstetrician. In order to standardise the method of suturing all episiotomies were repaired using the following standardized protocol: The vaginal wall was sutured continuously using Vicryl® 2-0 from the apex to the hymen. The bulbocavernosus and superficial perineal muscles and subcutaneous tissue were sutured with interrupted stitches using Vicryl® 0. The skin was sutured intracutaneously using Monocryl® 3-0 SH or Vicryl Rapide™ 3-0 SH. The first knot was tied in the subcutaneous tissue at the distal end of the episiotomy. From here a continuous intracutaneous suture to the fourchette was made where a knot was tied.

After randomization and perineal repair, women were handed out a questionnaire with four sets of questions to be answered 24 h, 10 days, six weeks and three months after delivery. Baseline characteristics were recorded from the hospital records. Pain scores were evaluated using a Visual Analogous Scale, (VAS), a self-reported pain scale from 0 to 10 cm [22]. VAS scores were evaluated in lying and sitting position, and whilst walking. Primary outcome was pain in sitting position after 10 days. Secondary outcomes were self-reported dehiscence, infections, analgesia use, necessity for removal of suture material, the resumption of intercourse and dyspareunia at 10 days, six weeks and three months. All the secondary outcomes were self-reported through the questionnaires. Women received text messages on day 10, week six and after three months as a reminder to fill out the particular part of the questionnaires. After three months they were asked to send back the questionnaires. Stamped return-envelopes were provided with the questionnaires. Non-responders were contacted by telephone and asked to send in the information (Additional file 1).

### Statistical analysis

Power analysis was performed prior to the study with the assumption of a difference in VAS score of 1 cm. With a α of 0.05 and a β of 90% it was calculated that two groups of 112 women were necessary to find statistically significant differences. Estimating 10% loss to follow up the total amount of inclusions was set to be 250.

Statistical analysis for the primary outcome and secondary pain scores was made using Student’s t-tests. Chi-square and Fishers exact test were used to investigate differences in proportions between independent categorical variables. Adjusted analyses were made using linear regression analysis and Mantel-Haenzel tests. No interim analyses were planned or performed. IBM SPSS Statistics for Windows version 22.0 was used for data analysis.

The trial was approved by the Ethical Committee for Scientific Research of Rotterdam (TWOR) ref. nr NL28922.101.10 on the 7th October 2010 and was registered retrospectively under number ISRCTN29869308.

## Results

Between November 2010 and July 2013 4995 women delivered in our hospital, with 2735 primiparous women who delivered vaginally. Over the research period the percentage of primiparous women with an episiotomy decreased from 52 to 47%.

Two-hundred-and-fifty women were randomized. Four inclusions were withdrawn. Three were erroneously included, one woman was not primiparous and two women did not meet the inclusion criterion of an uncomplicated mediolateral episiotomy. During repair one woman appeared to have a complicated vaginal wall rupture, whereas the other woman had a grade 3a rupture. For one woman two envelopes were opened accidentally, of which only one was used.

**Fig. 1:**
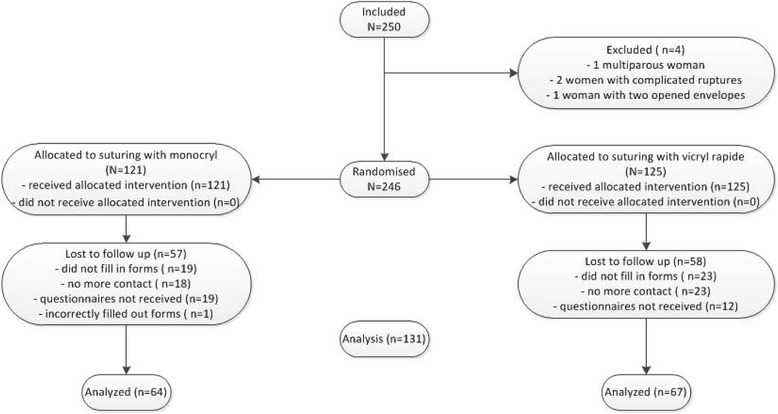
Flow chart MOVE trial

All women received the assigned treatment. Of the 246 included women 132 returned the questionnaires and one woman was excluded for analysis because she had not completed the VAS scores. Therefore, statistical analysis was performed on 131 women (53.3%) (Fig. 1).

Baseline characteristics of women in both groups showed no differences in terms of ethnicity, duration of second stage of labour and weight of the newborn between the two trial arms (Table 1). Women who were delivered by ventouse were more often allocated to Vicryl Rapide™. Due to the nature of the randomization method this unequal distribution occurred by chance.

**Table 1:** Baseline characteristics of the trial population

	Monocryl (*n* = 64)	Vicryl Rapide (*n* = 67)
Age (years ± SD)	31.5 ± 4.5	30.8 ± 4.4
Caucasian	57 (89%)	58 (87%)
Birth weight (gram ± SD)	3470 ± 361	3489 ± 500
Duration 2nd stage (minutes ± SD)	40 ± 29	39 ± 27
Direct Occiput Posterior Position	4 (6%)	2 (3%)
Ventouse delivery	7 (11%)	19 (28%)
Gestational age (weeks ± SD)	40 + 1 ± 8.9 days	40 + 1 ± 8.6 days
Apgar score 1 min (mean± SD)	9 ± 0.79	9 ± 0.80
Apgar score 5 min (mean± SD)	10 ± 0.83	10 ± 0.46

Continuous variables are given as means with standard deviations [SD]. Categorical variables are given as frequencies with percentages, *n* (%)

Responders were significantly older than non-responders and were significantly more often Caucasian. Other characteristics did not differ significantly between responders and non-responders (Table 2).

**Table 2:** Baseline characteristics responders vs. non-responders

	Reponders (*n* = 132)	Non-responders (*n* = 114)	*p*-value
Age (years, ± SD)	31,1 ± 4,5	28,4 ± 5.3	0.000
Caucasian	116 (88%)	82 (72%)	0.002
Birth weight (gram, ± SD)	3478 ± 438	3395 ± 508	0.17
Duration 2nd stage (minutes, ± SD)	39 ± 28	37 ± 28	0.49
Direct Occiput Posterior Position	6 (5%)	4 (4%)	0.69
Ventouse delivery	26 (20%)	23 (20%)	0.89
Gestational age (weeks, ± SD)	40 + 1 ± 8.6 days	40 + 1 ± 8.8 days	0.94
Apgar score 1 min (mean ± SD)	9 ± 0.79	9 ± 0.81	0.38
Apgar score 5 min (mean ± SD)	10 ± 0.66	10 ± 0.79	0.23

Continuous variables are given as means with standard deviations [SD]. Categorical variables are given as frequencies with percentages, n (%) and *p*-values

No significant difference was found in the primary outcome, pain (VAS score) in sitting position at 10 days after delivery. Furthermore, VAS scores did not show any statistically significant differences between the groups at any moment of comparison or in any position (Table 3).

**Table 3:** Primary outcomes

	Monocryl (*n* = 64)	Vicryl rapide (*n* = 67)	*p*-value
VAS* 24 h sitting	6.0 ± 2.4	5.9 ± 2.4	0.80
VAS 24 h walking	5.1 ± 2.5	4.9 ± 2.5	0.72
VAS 24 h lying down	3.9 ± 2.5	3.2 ± 2.3	0.09
VAS 10 days sitting	2.8 ± 2.5	2.5 ± 2.1	0.43
VAS 10 days walking	2.1 ± 2.1	2.4 ± 2.4	0.48
VAS 10 days lying	1.5 ± 1.9	1.2 ± 1.6	0.24
VAS 6 weeks sitting	0.5 ± 1.2	0.5 ± 1.3	0.73
VAS 6 weeks walking	0.5 ± 1.0	0.3 ± 1.0	0.24
VAS 6 weeks lying	0.3 ± 0.7	0.2 ± 0.8	0.74
VAS 3 months sitting	0.06 ± 0.2	0.09 ± 0.6	0.60
VAS 3 months walking	0.06 ± 0.2	0.09 ± 0.4	0.67
VAS 3 months lying	0.03 ± 0.2	0.03 ± 0.2	0.97

Variables are given as means with standard deviations (± SD) and *p*-values. *Visual Analogous Scale (0-10 cm)

Analysis of the secondary outcomes (Table 4) showed that women in the Monocryl® group reported significantly less skin dehiscence when compared with women in the Vicryl Rapide™ group after 10 days and within the first three months.

**Table 4:** Secondary outcomes

	Monocryl (*n* = 64)	Vicryl rapide (*n* = 67)	*p*-value
Analgesia use 24 h	22 (34%)	29 (43%)	0.30
Dehiscence 24 h	0 (0%)	1 (1%)	0.51
Analgesia use 10 days	5 (8%)	10 (15%)	0.20
Dehiscence 10 days	5 (8%)	15 (22%)	0.02
Infection 10 days	4 (6%)	2 (3%)	0.32
Removal of stitches 10 days	3 (5%)	8 (12%)	0.14
Intercourse 6 weeks	27 (42%)	29 (43%)	0.96
Painless intercourse 6 weeks	12 (19%)	11 (16%)	0.75
Intercourse 3 months	52 (81%)	52 (78%)	0.61
Painless intercourse 3 months	32 (50%)	39 (58%)	0.50
Dehiscence within 3 months	6 (10%)	17 (25%)	0.016
Stitches removal within 3 months	9 (11%)	8 (12%)	0.72
Infection within 3 months	4 (7%)	2 (3%)	0.37

Variables are given as frequencies with percentages, n (%) and *p*-values

No differences were found in intercourse at 10 days post partum or in analgesia use 6 weeks and 3 months post partum between the groups.

Women who delivered by ventouse delivery did not show more dehiscence compared to women with a spontaneous delivery. (Ventouse 15% vs. No ventouse 18%, *P* = 0.74). After correction for the difference in ventouse deliveries in the groups the difference in self-reported dehiscence was still statistically significant. (10 days *p* = 0.021, three months *p* = 0.017). After correction for the difference in ventouse deliveries between the Monocryl® and Vicryl Rapide™ group there was no difference in pain scores between the Monocryl® and Vicryl Rapide™ group at 10 days (*p* = 0.14). Similarly, there were no differences in adjusted pain scores at any other moment in time.

Women reporting dehiscence did not have statistically significant higher VAS scores at day 10 when compared with women who reported no dehiscence (VAS 3.1 (SD 2.4) vs. VAS 2,5 (SD 2.3), *p* = 0.33). After six weeks women reporting dehiscence (VAS 1.5 (SD 2,4)) reported statistically significant higher pain scores than women reporting no dehiscence (VAS 0,4 (SD 1,0)) *p* = 0.02.

Women who had suture material removed did not report more pain when compared with women who had no suture material removed (10 days VAS 3.2 (SD 2,8) vs. VAS 2,6 (SD 2,3), *p* = 0.42 and six weeks VAS 0.7 (SD 1,8) vs. VAS 0.5 (SD 1.1), *p* = 0.41).

Analgesia use and VAS score did not correlate strongly. Women who used analgesia at 24 h had VAS scores between 2 and 10 whereas women who did not use analgesia had VAS scores between 0 and 10. The correlation coefficient was 0.10, with a *p* = 0.22 this was not significant. After 10 days women using analgesia had VAS scores between 1 and 8 and women who did not use analgesia had VAS scores between 0 and 9. At day 10 the correlation coefficient was 0.22 and although at a *p* = 0.01 this was significant, the correlation was weak. After 6 weeks and 3 months all women but one had stopped using analgesia (Additional file 2).

## Discussion

Our trial showed no difference in pain scores comparing Monocryl® and Vicryl Rapide™ for intracutaneous closure of the skin at the time of primary repair of uncomplicated mediolateral episiotomies in primiparous women. However, our trial did show significantly more self-reported dehiscence in the Vicryl Rapide™ group as compared to the Monocryl® group.

Until now only two trials compared a monofilament and a multifilament resorbable synthetic material for the repair of episiotomies. Dencker et al. [15] compared suturing of all wound layers of both perineal tears and episiotomies with a monofilament, glycomer 631, with polyglycolic acid, a multifilament suture material. Three days after repair no difference in wound healing was found but after eight weeks healing complications or necessity to remove suture material was reported significantly more often in the monofilament group (OR 1.62, 95% CI 1.04-2.54, *p* = 0.034). VAS scores higher than two at 8-12 week follow up were reported more often in the monofilament group (RR 1.51, 95% CI 1.01-2.24, *p* = 0.046). These results may be biased however, as both intracutaneous and interrupted techniques for skin closure were used. Due to external knots on the perineal skin, interrupted sutures may cause the painful so-called “barbed wire effect” [23]. It could be hypothesised that external knots of the monofilament suture material cause more complaints than the multifilament suture material. As the techniques were not compared, the influence of the technique on the outcome is unclear.

Kokanali et al. [16] compared polyglycolide-co-caprolactone, a monofilament material, with polyglactine 910 rapide with regard to healing and pain scores in women with a mediolateral episiotomy. With a continuous intracutaneous closing technique no differences in pain scores were found between both groups at 24 h (VAS 5.4 vs. VAS 5.7) and 10 days (VAS 2.7 vs. VAS 3.0) after delivery.

We found significantly more frequent self-reported dehiscence in the Vicryl Rapide™ group. These results may put into question whether rapidly absorbable multifilament synthetic material is the optimal material for perineal repair, as suggested in current guidelines [24].

In contrast to this, Dencker et al. [15] reported no difference in dehiscence between monofilament and multifilament suturing material at 8-12 weeks. The percentage of dehiscence found in the Vicryl Rapide™ 3-0 group was higher than that found in a similar group after the use of Vicryl Rapide™ 2-0 [25]. This could possibly be caused by the caliber of the thread but could also be attributed to the low response rate, which could overestimate the complications in our trial due to biased response.

A recent report suggested that resuturing of episiotomies in which dehiscence took place is beneficial for the short-term complaints of women. [26] The clinical significance of the self-reported dehiscence in our trial is unclear. Women with dehiscence did report a higher pain score after six weeks compared with those who did not. Whether women with pain had a lower threshold from where to report dehiscence is unclear. To conclude on this issue physical examination should be included in the trial.

In our trial 42-43% and 78-82% of women had resumed intercourse after six week and three months respectively, of which 41-48% and 68-80% without pain. There was no statistically significant difference between suture materials. These results were similar to those in some of the previous trials [7, 11] in which 33-66% of women had resumed intercourse at 6-7 weeks and 88-98% 3 months after episiotomy or 2nd degree perineal rupture. Necesalova et al. [6] found after 3 months 51% had no or rarely complaints of dyspareunia, after 6 months this was 68%. Dyspareunia rates found in this trial are hard to compare to ours because of a different way of questioning.

Loss to follow up was greater than expected in our trial. Loss to follow up at 6-12 weeks in comparable trials varies considerably between trials, from 3.3% to 64% [9, 27].

In our trial all questionnaires were integrated in a booklet and were given directly after delivery Additionally, participants received text-messages as reminders to fill out the questionnaires on day 10, six weeks and three months after delivery. Despite phone calls 46% of the questionnaire-booklets were never returned. Kettle [9] and Mahomed [28] sent questionnaires at three and/or 12 months instead of handing the questionnaire out after delivery, leading to 96% and 87% follow-up rate respectively. This approach may be more favourable to prevent loss to follow up.

In our trial non-responders were on average younger and the group contained less Caucasian women. Other characteristics were similar between the responder and non-responder group. Correcting for age and Caucasian background the outcome for VAS score at 10 days and dehiscence remained the same. Therefore we do not believe that the high number of women lost to follow up resulted in a bias in favour of one of the two used materials.

### Strength

The main strength of our study is that we aimed to find the best material for skin closure for a frequently used but under-researched operative procedure, the episiotomy. Episiotomy and its repair often cause complaints of pain and dyspareunia in women in the post partum period. Our trial is the third randomized controlled trial comparing a monofilament and a fast-absorbing multifilament suture material for episiotomy repair after Dencker et al. [15] and Kokanali et al. [16]. None compared the materials used in our study. We tried to minimize variance by including only primiparous women with uncomplicated episiotomies and using a standardized technique of repair.

### Limitations

One limitation was the low response rate in our trial. Fifty-four percent of the questionnaires were returned. As previously described we do not believe this resulted in biased results.

Variance of Visual Analogous Scores within both groups was large, despite efforts to maintain homogeneity. It is a well-known phenomenon that there is difference in pain perception among individuals and correlation between pain scores and analgesia use in our study was weak.. Due to the large variance, use of VAS scores might not be optimal in similar trials, since this requires large numbers of participants.

Dehiscence was self-reported and we did not perform clinical examination during the follow up period. The clinical significance of the dehiscence is not clear. In our trial we did not ask about complaints apart from pain. VAS scores after 6 weeks in women who reported dehiscence were higher than in women who did not.

## Conclusions

In our trial, pain after episiotomy repair did not differ between skin suturing with Monocryl® or Vicryl Rapide™. Pain scores after mediolateral episiotomy repair were generally low after 6 weeks and 3 months postpartum. Our trial is the first to report a significant difference in dehiscence in favour of Monocryl® compared to Vicryl Rapide™ over the three-month study period. As pain is comparable between the two materials, Monocryl® may therefore be slightly superior over Vicryl Rapide™ when suturing the skin intracutaneously in mediolateral episiotomies. It has to be stated however that the clinical significance in the difference of the self-reported dehiscence remains to be determined. Further trials, preferably with clinical examinations at set time points in the postpartum period, are necessary to support these results.

## Additional files

Additional files 1: Questionnaire as handed out to participants of the MOVE Trial. (DOCX 399 kb)

Additional files 2: Baseline characteristics of participants and primary and secondary outcomes of the trial. (XLS 154 kb)

## Abbreviations

VAS: Visual Analogous Scale
