# Supplementary material for: The MOVE-trial: Monocryl® vs. Vicryl Rapide™ for skin repair in mediolateral episiotomies: a randomized controlled trial
Source: BMC Pregnancy Childbirth. 2017 Oct 16;17:355. doi: 10.1186/s12884-017-1545-8 (PMC5644141; doi:10.1186/s12884-017-1545-8)
Supplement: Supplementary file 1 — Questionnaire as handed out to participants of the MOVE Trial. (DOCX 399 kb) [file 12884_2017_1545_MOESM1_ESM.docx]

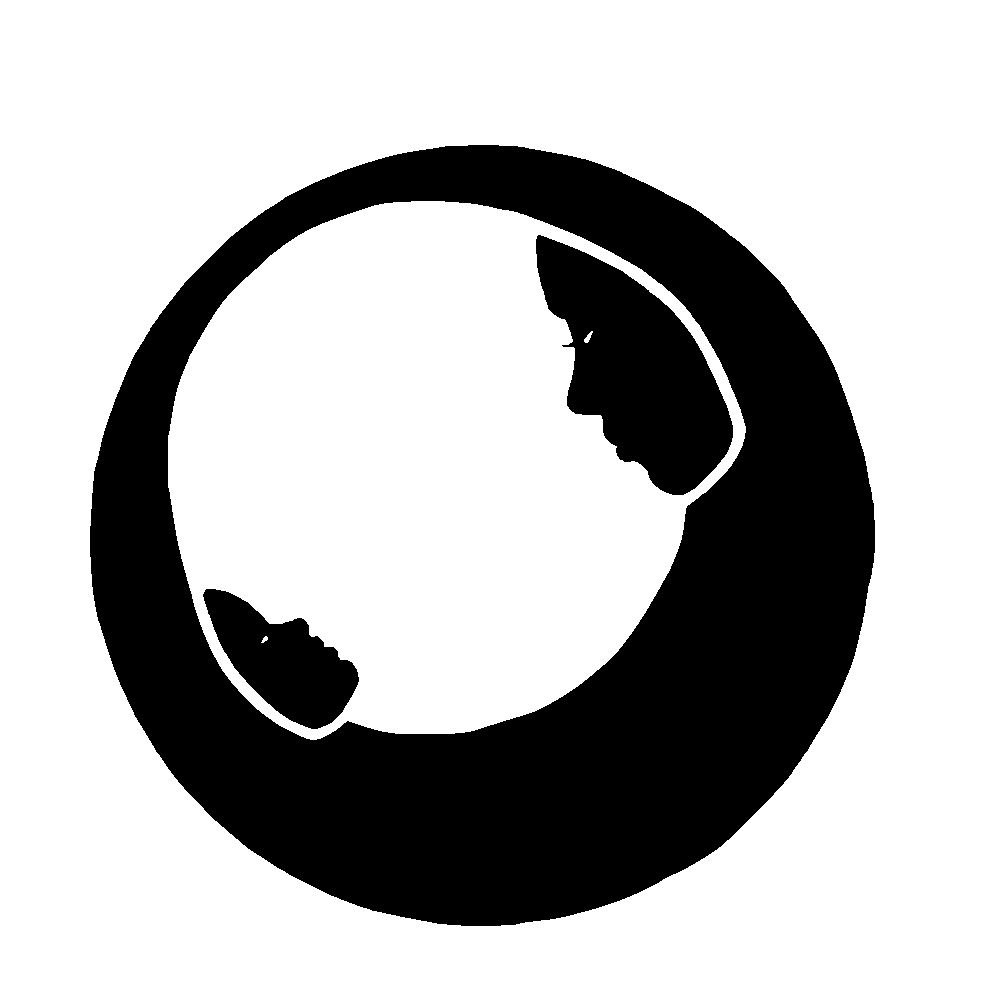


M VE


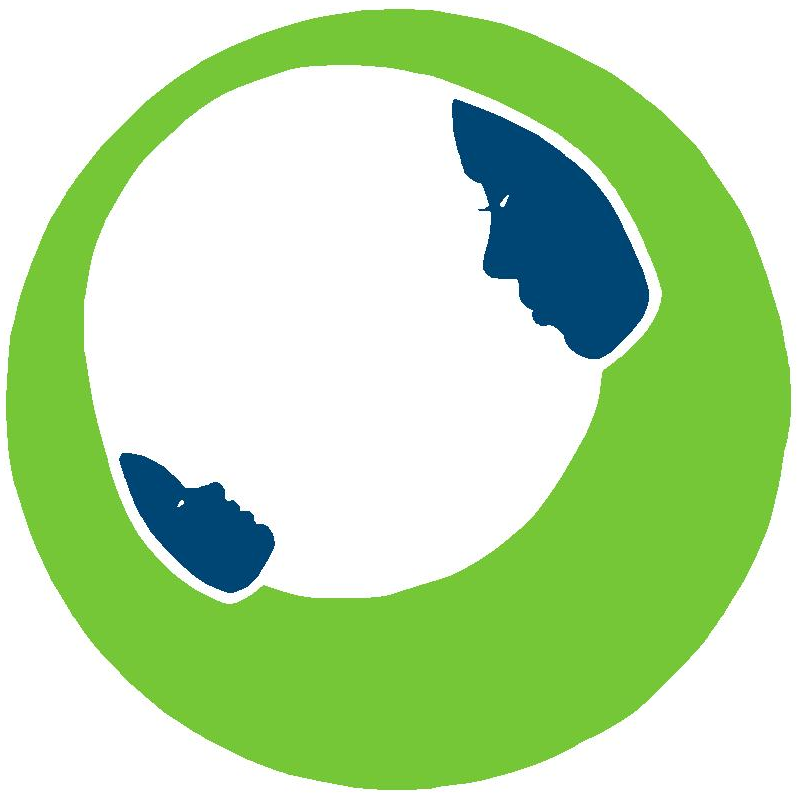


Questionnaire

Research number:

Dates on which to fill out the questionnaire:

First list (12-24 hours) : .............................


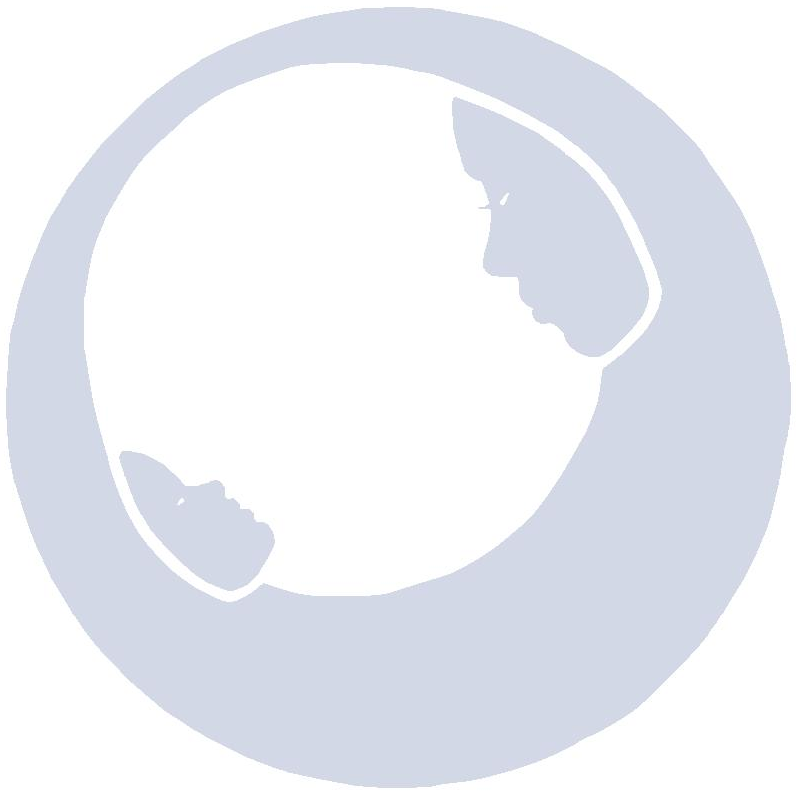
Second list (10 days) : .........................

Third list (6 weeks) : .............................

Fourth list (3 months) : ........................

**De MOVE–Trial.**

**Dear Madam.**

**You participate in the MOVE-trial. During your delivery there has been a reason to perform an episiotomy. In this trial we wish to discover which is the optimal material for suturing the episiotomy. In order to do this we ask you to fill out four short questionnaires. After we compare questionnaires of participants we hope to be able to determine which material we should use for suturing in the future.**

**We ask you to fill out the first questionnaire when you are still at the hospital, 12 to 24 hours after delivery. The other questionnaires can be filled out at home. This should only take a few minutes.**

**As a reminder, you will receive a tekst message to fill out the questionnaire on the day the questionnaire should be completed.**

**Once you have completed the last questionnaire, after 3 months, we ask you to return the questionnaires to us using the enclosed envelope. Then we can start the analysis of the data.**

**We would like to thank you in advance for your co-operation. Without your participation we would not be able to perform this research.**

**In case you have any questions or comments, please contact one of the medical specialists stated below at any time.**

**With kind regards,**

**1st Contact**

**Roeland Odijk, Arts assistent Gynaecologie, onderzoeker**

[movestudie@gmail.com](mailto:movestudie@gmail.com)

**via polikliniek gynaecologie, 010-2975240**

**2nd Contact**

**Mw. A.C. van Hof, Gynaecoloog**

**Via polikliniek gynaecologie, 010-2975240**

**3rd Contact**

**Dr. W.J. Vles, Chirurg**

**Via secretariaat chirurgie 010-2975365**

**12 – 24 hours after delivery**

**Date:**

**MOVE-study : Pain complaints after suture of an Episiotomy**

Dear Madam

It’s less than 24 hours after your delivery.

This is the first questionnaire of a series of questionnaires that we would like to ask you to complete the coming months. Right now, you probably are still at the hospital. You can take the questionnaires home, complete them at the appropriate time and then send them to us in the enclosed envelope after three months.
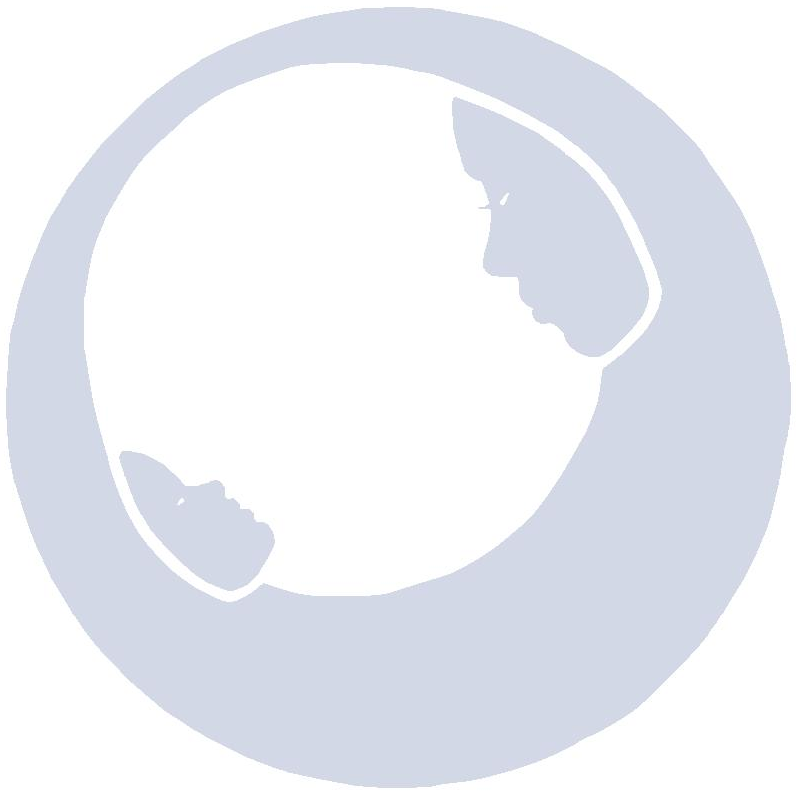


**Research number:**

How much pain do you experience in sitting position? Please rate from 0 (no pain) to 10 (unbearable pain)

None -------------------------------------------------------------------------------------- Maximum

How much pain do you experience walking? Please rate from 0 (no pain) to 10 (unbearable pain)

None -------------------------------------------------------------------------------------- Maximum

How much pain do you experience lying down? Please rate from 0 (no pain) to 10 (unbearable pain)

None -------------------------------------------------------------------------------------- Maximum

Did you use pain medication today? Yes / No*

If yes, which (kind and doses) and how many?........................................................................

Did the wound open up? Yes / No*

Did you experience other problems with the suture? Yes / No*

If yes, which one(s)?

- Delete as appropriate.

**10 days after delivery**

**Date:**

**MOVE-study : Pain complaints after suture of an Episiotomy.**

Dear madam

It has been 10 days since you gave birth.

We would like to ask you to fill out this questionnaire today.


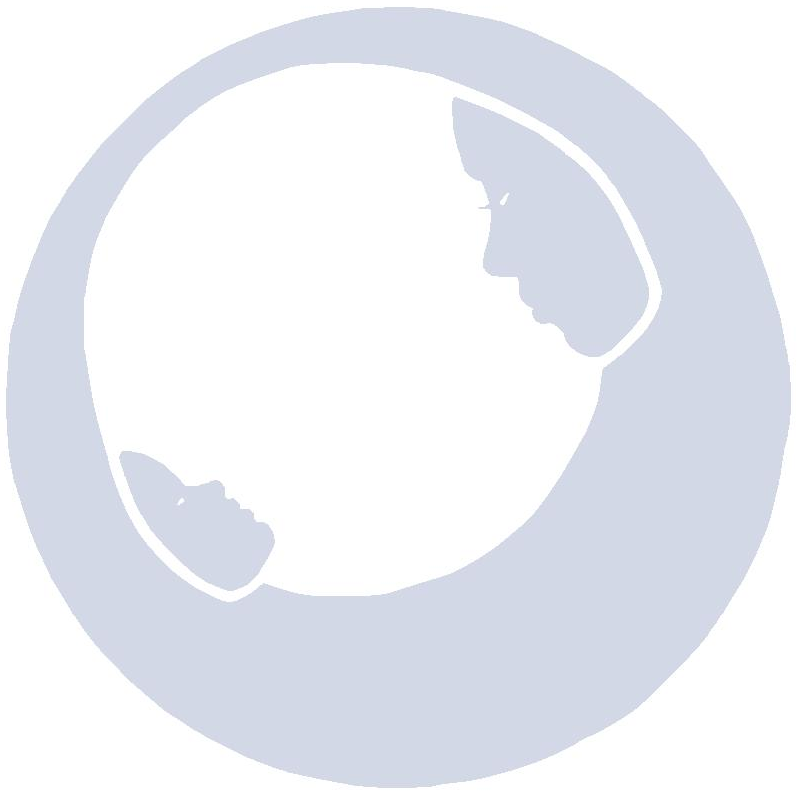


How much pain do you experience in sitting position? Please rate from 0 (no pain) to 10 (unbearable pain)

None -------------------------------------------------------------------------------------- Maximum

How much pain do you experience walking? Please rate from 0 (no pain) to 10 (unbearable pain)

None -------------------------------------------------------------------------------------- Maximum

How much pain do you experience lying down? Please rate from 0 (no pain) to 10 (unbearable pain)

None -------------------------------------------------------------------------------------- Maximum

Did you use pain medication today? Yes / No*

If yes, which (kind and doses) and how many?........................................................................

Did you have an infection at the location of the suture? Yes / No*

Did someone remove the stitches? Yes / No*

Did the wound open up? Yes / No*

* Delete as appropriate.

**MOVE-study : Pain complaints after suture of an Episiotomy ( 10 days)**

Did you have sexual intercourse since the delivery? Yes / No*

If not, was this due to pain? Yes / No*


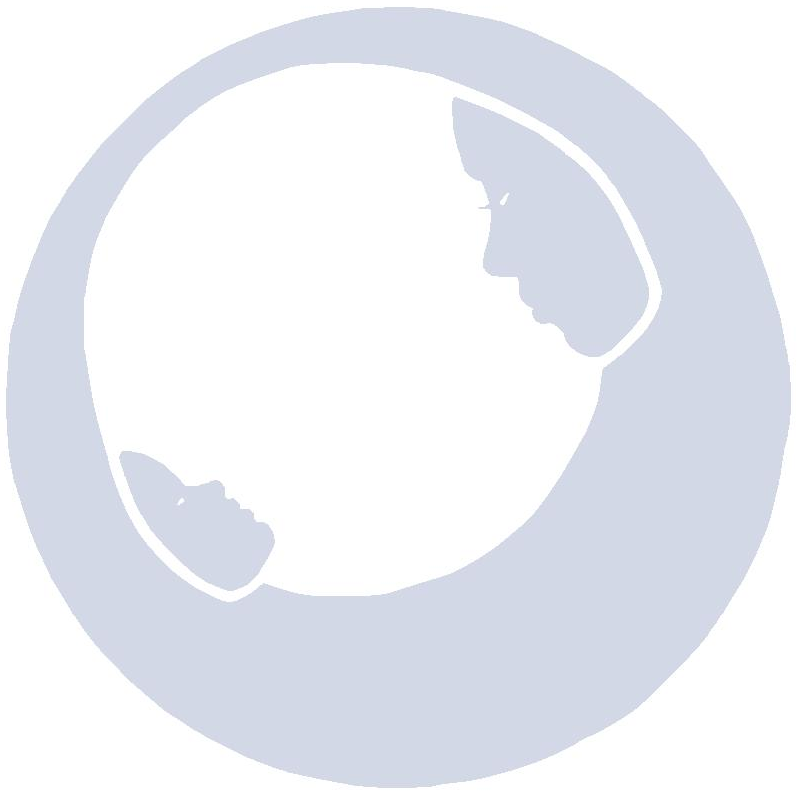


If yes, how much pain do you currently experience on average during sexual intercourse?

None -------------------------------------------------------------------------------------- Maximum

Have you had pain-free sexual intercourse since delivery? Yes / No*

Did you experience other problems with the suture? Yes / No*

If yes, which one(s)?

Please state any additional remarks:……………

* Delete as appropriate.

**6 weeks after delivery**

**Date:**

**MOVE-study : Pain complaints after suture of an Episiotomy**

Dear Madam

It has been 6 weeks since you gave birth.

We would like to ask you to fill out this questionnaire today.


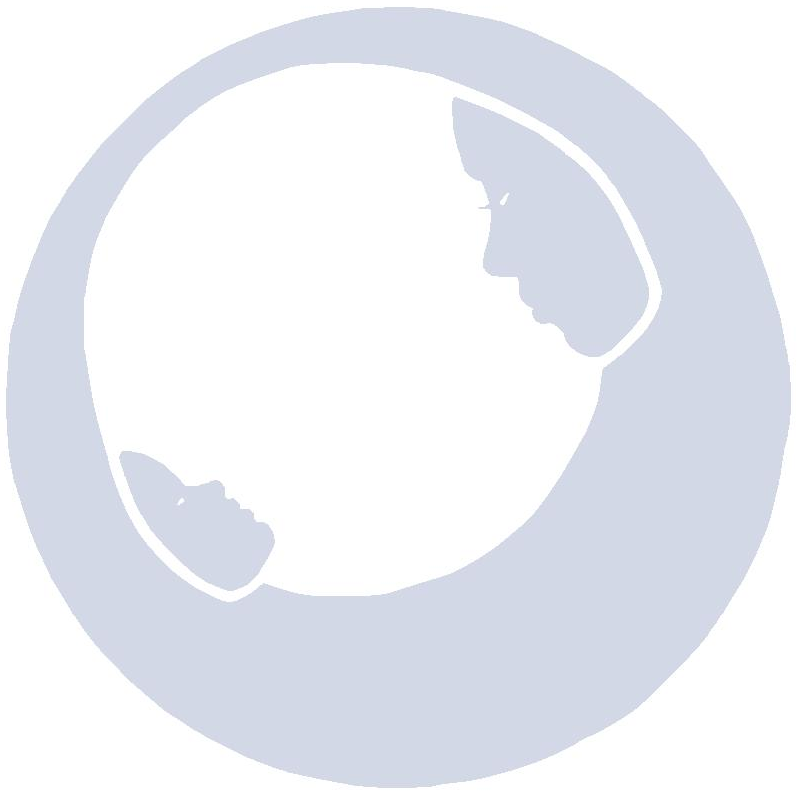


How much pain do you experience in sitting position? Please rate from 0 (no pain) to 10 (unbearable pain

None -------------------------------------------------------------------------------------- Maximum

How much pain do you experience walking? Please rate from 0 (no pain) to 10 (unbearable pain)

None -------------------------------------------------------------------------------------- Maximum

How much pain do you experience lying down? Please rate from 0 (no pain) to 10 (unbearable pain)

None -------------------------------------------------------------------------------------- Maximum

Did you use pain medication today? Yes / No*

If yes, which (kind and doses) and how many?........................................................................

Did you have an infection at the location of the suture? Yes / No*

* Delete as appropriate.

**MOVE-study : Pain complaints after suture of an Episiotomy. (6 weeks)**

Did someone remove the stitches? Yes / No*

If so, why?............................................................................

Did the wound open up? Yes / No*


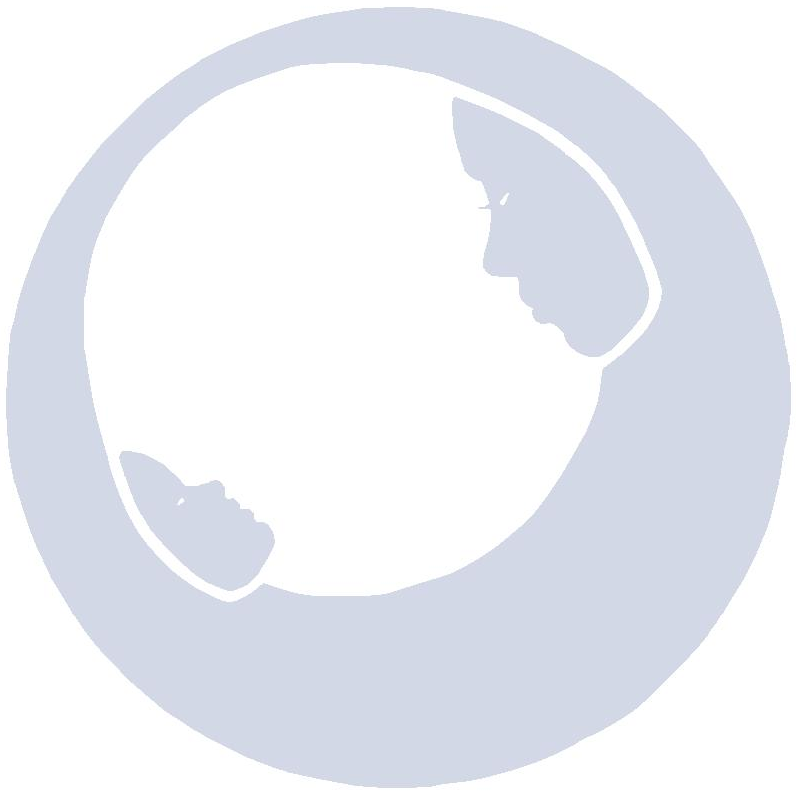


Did you have sexual intercourse since the delivery? Yes / No*

If not, was this due to pain? Yes / No*

If yes, how much pain do you currently experience on average during sexual intercourse?

None -------------------------------------------------------------------------------------- Maximum

Have you had pain-free sexual intercourse since delivery? Yes / No*

Did you experience other problems with the suture? Yes / No*

If yes, which one(s)?

Please state any additional remarks:……………

* Delete as appropriate.

**3 months after delivery**

**Date:**

**MOVE-study : Pain complaints after suture of an Episiotomy**

Dear Madam

It now has been 3 months since you gave birth.

We would like to ask you to fill out this questionnaire today. Please return the questionnaires to us once you are finished.


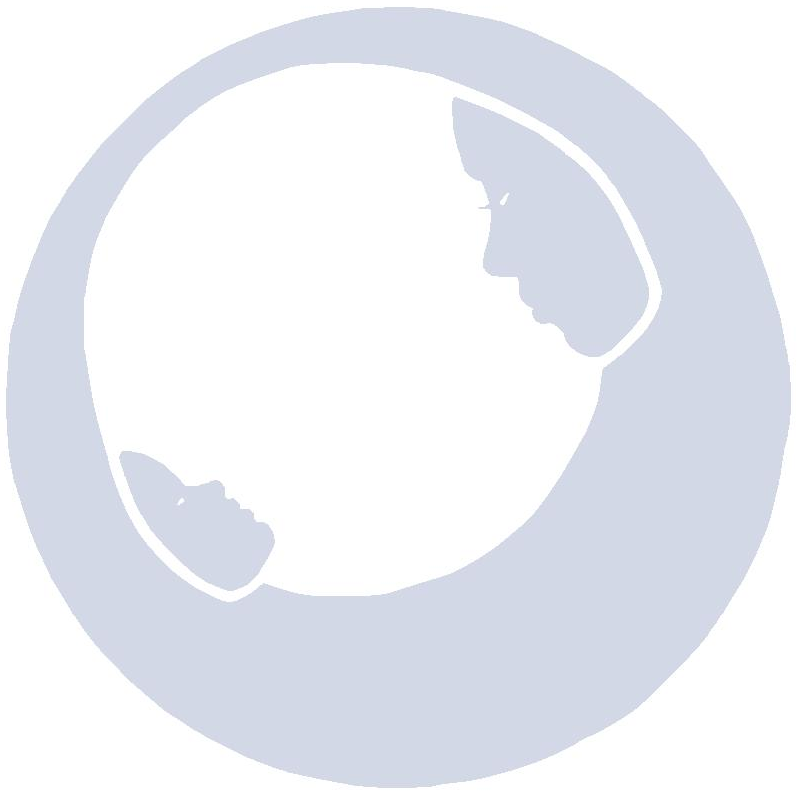


How much pain do you experience in sitting position? Please rate from 0 (no pain) to 10 (unbearable pain)

None -------------------------------------------------------------------------------------- Maximum

How much pain do you experience walking? Please rate from 0 (no pain) to 10 (unbearable pain)

None -------------------------------------------------------------------------------------- Maximum

How much pain do you experience lying down? Please rate from 0 (no pain) to 10 (unbearable pain)

None -------------------------------------------------------------------------------------- Maximum

Did you use pain medication today? Yes / No*

If yes, which (kind and doses) and how many?........................................................................

Did you have an infection at the location of the suture? Yes / No*

* Delete as appropriate.

**MOVE-study : Pain complaints after suture of an Episiotomy (3 months)**

Did someone remove the stitches? Yes / No*

If so, why?............................................................................

Did the wound open up? Yes / No*


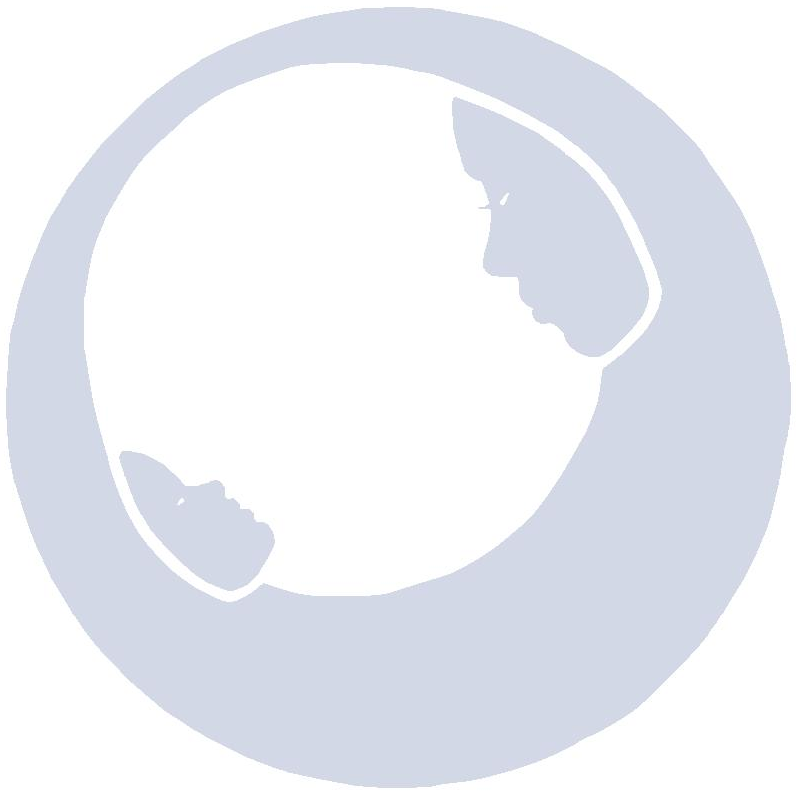
Did you have sexual intercourse since the delivery? Yes / No*

If not, was this due to pain? Yes / No*

If yes, how much pain do you currently experience on average during sexual intercourse?

None -------------------------------------------------------------------------------------- Maximum

Have you had pain-free sexual intercourse since delivery? Yes / No*

Did you experience other problems with the suture? Yes / No*

If yes, which one(s)?

Please state any additional remarks:……………

* Delete as appropriate.

**After filling out this last page we would like to ask you to return the questionnaire in the enclosed envelope.**

**We would like to thank you for your cooperation.**
